# The efficacy of wound catheter infusion with local anesthetics for the treatment of postoperative pain in children: A systematic review

**DOI:** 10.1002/pne2.12126

**Published:** 2024-06-05

**Authors:** Dominique J. Swenker, Maaike Dirckx, Lonneke M. Staals

**Affiliations:** ^1^ Department of Anesthesiology, Erasmus MC University Medical Center Rotterdam Rotterdam The Netherlands; ^2^ Department of Anesthesiology, Erasmus MC Sophia Children's Hospital University Medical Center Rotterdam Rotterdam The Netherlands

**Keywords:** analgesia, child, local anesthetic, opioid analgesics, pain management, wounds

## Abstract

Wound catheter infusion (WCI) with local anesthetics (LA) is a regional anesthesia technique, which has shown to produce effective postoperative analgesia in adults, without any adverse effects on wound healing. To investigate the efficacy and safety of WCI with LA for the treatment of postoperative pain in children, we conducted a systematic review of literature published until 2020. The literature search included articles concerning subcutaneous WCI with LA, in the surgical wound, as treatment of postoperative pain, in children <18 years of age. Exclusion criteria were studies describing peripheral nerve blocks, intercostal, abdominal or thoracic wall blocks and single local anesthetic infiltration of the surgical wound. The articles were appraised for quality and only randomized controlled trials with a Jadad score ≥3 were included for evaluation of results concerning postoperative pain scores and opioid use. All relevant original studies, including observational studies and case reports, were assessed for adverse events and measurements of LA plasma concentrations during WCI. A total of 1907 articles were found, leading to 92 relevant abstracts selected for further review. After exclusion of articles of which full texts could not be retrieved or because of exclusion criteria, 28 articles remained. Thirteen articles described randomized controlled trials, of which 10 were assessed as good or excellent in quality. Due to the small number and heterogeneity of the studies, the data could not be pooled. Instead, results were described per type of procedure: abdominal surgery, extremity surgery, thoracic surgery and iliac crest bone harvesting. Reduced pain scores and opioid needs were demonstrated after abdominal and extremity surgery. In five studies, plasma levels of LA were measured, which all remained below toxic thresholds. In all relevant studies, no serious adverse events concerning the use of WCI were reported.

## 1 INTRODUCTION

Subcutaneous wound catheter infusion (WCI) with local anesthetics (LA) is a regional anesthesia (RA) technique, which has shown to produce effective postoperative analgesia in adults. At rest, analgesia was comparable to epidural analgesia.^1^ The catheter is placed in the subcutaneous tissue along the length of the surgical wound, prior to closure of the skin, and used to infiltrate the wound with LA, either continuously or with intermittent boluses.^1^ WCI was associated with reduced pain scores, decreased opioid requirements, reduced length of stay and was not associated with any adverse effects regarding wound healing.^1^, ^2^

In children, the use of WCI is relatively new. Neuraxial and regional blocks are performed widely in pediatric anesthesia. Although these techniques are considered safe, still, in neuraxial blocks complications such as dural puncture and transient neurological deficits may occur.^3^, ^4^, ^5^ WCI could be an effective alternative for treatment of postoperative pain.

If the use of WCI with LA could lead to a reduction in intravenous (IV) opioids, side effects of opioids could be reduced, such as respiratory depression, but also nausea, vomiting and urinary retention.^6^

We conducted a systematic review to evaluate the analgesic effect and safety of WCI in children. The main goal was to assess the efficacy of the WCI technique with LA for the treatment of postoperative pain in children, as measured by postoperative pain scores and effect on opioid consumption, and if so, for which kind of procedures WCI would be beneficial.

## 2 METHODS

The study was registered in Prospero (CRD42021248594) and the manuscript adheres to the applicable PRISMA guidelines.

### 2.1 Literature search

In 2015, a literature search was performed, according to the following search criteria: “children”, “wound catheter”, “continuous infusion” of “LA” and “postoperative pain” (full search terms—MeSH—list in Appendix A1). The search was conducted in Embase, Medline (Ovid‐SP), Cochrane Central, Web of Science and Pubmed Recent. The search then yielded only 14 relevant articles, with varying quality and describing heterogeneous patient groups. Therefore, in 2020 an update of the search was requested, using the same search criteria and search engines. In 2020, the search was not performed in PubMed Recent, as these articles are nowadays also included in Medline (Ovid‐SP). Articles were presented to the authors by the Biomedical Information Specialist of the medical library, using Endnote Library, after elimination of duplicates.

### 2.2 Screening of titles and abstracts

Abstracts of articles found in 2015 and of the updated search in 2020 were screened independently by LS and DS, to see if the articles met the search criteria: children (0–18 years old), receiving WCI with LA, as continuous or intermittent administration in the surgical wound (subcutaneously), for the treatment of postoperative pain. Articles concerning “adult patients”, “nonhumans”, describing “peripheral nerve blocks”, “intercostal”, “abdominal wall” or “thoracic wall blocks”, infusion in “peritoneum” or “fascial plane”, “single infiltration” of the wound with LA, non‐original studies like reviews and expert opinions and articles written in another language than English were excluded. No distinction was made what WCI was compared to, all compared techniques were noted. Any discrepancies in the final selection were discussed by two authors (DS and LS). If the suitability of the article was still in doubt, it was included. The full texts of the publications thus selected were obtained. If the full text could not be retrieved, or if by reading the full text it became clear the article did not meet the inclusion criteria, the article was excluded. The references of all thus selected articles were checked to see if any other relevant papers had been missed.

### 2.3 Main and additional outcomes

Primary outcome was efficacy of WCI with LA for the treatment of postoperative pain: effect on pain scores and need for analgesics postoperatively. To assess the primary outcome, only randomized controlled trials (RCTs) of good or excellent quality were included for further analysis. The RCTs were assessed for validity and quality independently by the three authors (DS, LS and MD), using the Jadad score.^7^ Any discrepancies were resolved after mutual evaluation and discussion. Of RCTs with a Jadad score of three and higher (good or excellent), the primary outcome was noted. Also the following information was gathered; total number of patients, patient age, type of surgery, type and dose of LA used, what comparison was used, which pain assessment tool was used, if this tool was validated for the age group investigated, and if any other outcomes were presented.

Secondary outcome of this investigation was safety of WCI with LA in children. All relevant studies were evaluated for adverse events concerning the use of WCI with LA, such as accidental overdose of or allergic reaction to LA, symptoms of LA systemic toxicity (LAST), improper wound healing, occurrence of infection, bleeding, inadvertent early catheter displacement, and any other malfunction of the catheter system. In addition, it was noted if pharmacokinetic data were collected.

### 2.4 Analysis of the data

RCTs with a Jadad score of three and higher were analyzed per type of surgery, and if possible by age group (neonates, infants, children, adolescents). Data was planned to be pooled describing postoperative treatment for one type of surgery, using WCI with one type of local anesthetic, compared to control patients. As we expected a low number of eligible studies, no quantitative synthesis or statistical test would be performed. For the results on adverse events, we pooled the data describing adverse events in all eligible studies, and compared the patients receiving WCI with LA with all control patients.

## 3 RESULTS

### 3.1 Inclusion of articles

**FIGURE 1:**
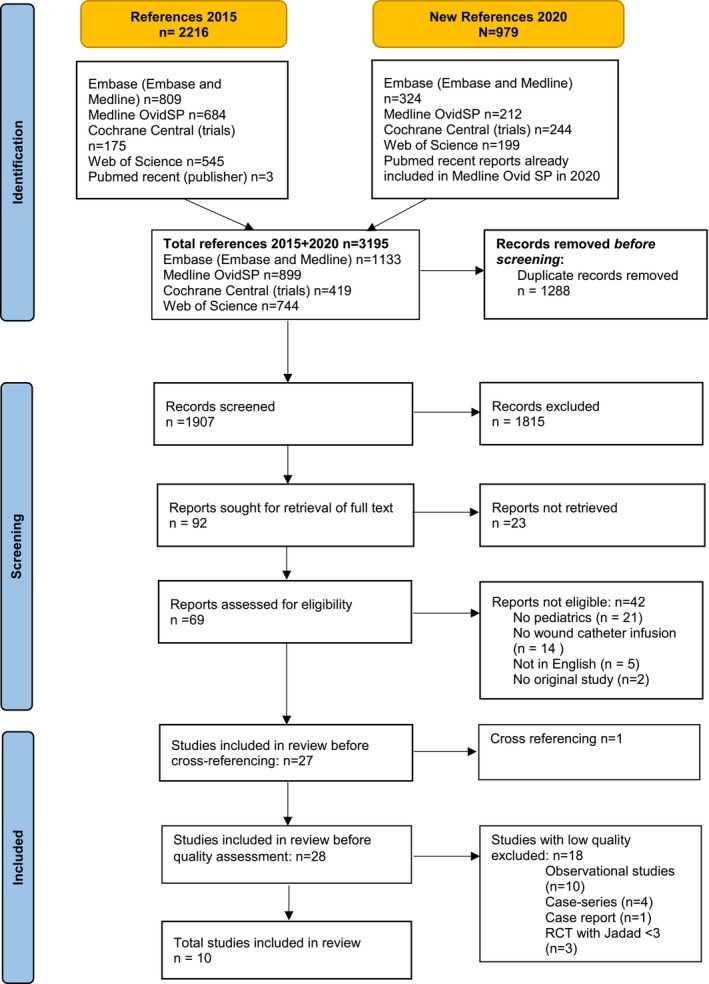
Search strategy, presented in a flow diagram according to the PRISMA guidelines. RCT, randomized controlled trial.

The first literature search was conducted on February 5, 2015. It was repeated on the July 31st 2020. Figure 1 shows the PRISMA flow diagram of the identification of articles and screening process. In total, the search yielded 1907 titles after elimination of duplicates (683 new titles in 2020). After screening title and abstract, 92 articles were selected for further review. Of 23 articles no full text was available (e.g., either registration of a study which is still being enrolled or abstract presentation at congress), and 42 articles were excluded. The remaining articles were checked for cross‐references and one relevant article was found and included.^8^

The final 28 articles consisted of 13 RCTs,^8^, ^9^, ^10^, ^11^, ^12^, ^13^, ^14^, ^15^, ^16^, ^17^, ^18^, ^19^, ^20^ 10 observational studies,^21^, ^22^, ^23^, ^24^, ^25^, ^26^, ^27^, ^28^, ^29^, ^30^ four case‐series^31^, ^32^, ^33^, ^34^ and one case‐report.^35^ These articles were assessed for information regarding the safety of WCI with LA in children.

Table 1 shows the RCTs Jadad scores. Three RCTs with a Jadad score <3 (poor quality) were excluded.^10^, ^12^, ^18^ The 10 RCTs with good or excellent quality (Jadad score ≥3) remained for analysis of the primary outcome.^8^, ^9^, ^11^, ^13^, ^14^, ^15^, ^16^, ^17^, ^19^, ^20^

**TABLE 1:** Jadad scores of included RCTs.

Study reference								
Item no.	1	2	3	4	5	6	7	Total
Anell‐Olofsson et al.^9^	1	1	1	1	1	0	0	5
Bulut et al.^8^	1	1	1	0	1	0	0	4
Hayes et al.^10^	1	0	0	1	0	0	0	2
Hermansson et al.^11^	1	1	1	1	1	0	0	5
Hidas et al.^12^	1	0	0	1	0	0	0	2
Kumar Raja et al.^13^	1	1	1	1	0	0	0	4
Machoki et al.^14^	1	0	1	1	0	0	0	3
Mattila et al.^15^	1	1	0	1	1	0	0	4
Meara et al.^16^	1	1	0	1	1	0	0	4
Muthusamy et al.^17^	1	0	1	1	0	0	0	3
Niiyama et al.^18^	1	0	1	0	0	0	0	2
Samartzis et al.^19^	1	1	1	1	1	0	0	5
Tirotta et al.^20^	1	1	1	0	0	0	0	3

*Note*: Score interpretation: 0–2 poor, 3–4 good, 5 excellent. How items were scored using the Jadad score^7^: Either give a score of 1 point for each “yes” or 0 points for each “no”. There are no in‐between marks. (1). Was the study described as randomized (this includes the use of words such as randomly, random and randomization)? (2). Was the study described as double blind? (3). Was there a description of withdrawals and dropouts? (4–7). Give 1 additional point if: For question 1, the method to generate the sequence of randomization was described and it was appropriate (table of random numbers, computer generated, etc.) And/or: If for question 2 the method of double blinding was described and it was appropriate (identical placebo, active placebo, dummy, etc.) Deduct one point if: For question 1, the method to generate the sequence of randomization was described and it was inappropriate (patients were allocated alternately, or according to date of birth, hospital number, etc.) And/or: For question 2, the study was described as double blind but the method of blinding was inappropriate (e.g., comparison of tablet vs. injection with no double dummy).

### 3.2 Efficacy of WCI

The selected studies described heterogeneous patient groups, types of surgery and types and concentrations of LA. The results are described per type of surgery in the following text. Table 2 presents the included studies in detail: patient characteristics, LA administered (including dosing regimens), comparator, outcomes, and adverse events.

**TABLE 2:** Results of RCTs on WCI in children, with a Jadad score ≥3 (good or excellent quality).

Article	Patients	Surgery	Local anesthetic	Control group	Outcome measurement tool	Conclusion	Adverse events
*Thoracic surgery*							
Anell‐Olofsson et al.^9^ 2015	9 WCI, 9 control. Gestational age 26 + 0–33 + 2 Weight 642‐1650 g	Closing patent ductus arteriosus via posterolateral thoracotomy	Levobupivacaine 0.0625% Bolus 0.2 mg/kg Continuous levobupivacaine 2.0 mg/kg/24 h. Switch to saline 8 h postoperatively. The infusion was switched every 8 h between saline and levobupivacaine. Duration 24 h.	Bolus with saline. First WCI with saline for 8 h, then switch to levobupivacaine 2.0 mg/kg/h. The infusion was switched every 8 h between saline and levobupivacaine.	Echelle doulour inconfort nouveau (minimum 0 points, maximum 15 points)	Median pain scores study group 0, control group 1, no significant difference. More IV fentanyl as rescue analgesia in control group (*n* = 7 vs. *n* = 12). More reduction of morphine infusion in study group (*n* = 6 vs. *n* = 1).	*N* = 1 wound infection *N* = 8 fluid leakage
Mattila et al.^15^ 2016	26 WCI, 23 control. Age 1.3–9.7 years Mean weight 18.6 kg	Atrial septal defect closure	Ropivacaine 0.2% Continuous 0.3–0.4 mg/kg/h Duration 47–54 h	WCI with saline	Objective Pain Scale	Pain scores and morphine consumption similar	*N* = 1 catheter dislodgement
Tirotta et al.^20^ 2009	35 WCI, 37 control Age 3–200 months Weight 5.1–93 kg	Cardiac surgery via median sternotomy	Bupivacaine 0.25% or levobupivacaine 0.25% Continuous 0.16–0.40 mg/kg/h Duration 64‐135 h	WCI with saline	FLACC, NRS, Wong‐Baker FACES Pain rating Scale	No difference in pain scores. Less total IV morphine over 1st 24 h in treatment group (0.05 mg/kg vs 0.2 mg/kg). No significant difference day 2 and 3	*N* = 1 catheter luxation
*Iliac crest bone harvesting*							
Kumar Raja et al.^13^ 2014	20 WCI, 20 single subcutaneous infiltration LA, 20 femoral nerve block Mean age 9,7 years	Unilateral secondary alveolar bone grafting with bone from anterior iliac crest	Bupivacaine 0.5% Bolus 1–1.5 mg/kg Repeated on request as rescue Duration 48 h	20x infiltration of 2 mL bupivacaine 0.5% + WC with bolus” of saline on request as rescue20x preoperative single‐shot femoral nerve block + WC with bolus” of saline on request as rescue	Wong‐Baker FACES rating scale	Lower pain scores (4.0 ± 0.67 vs. 6.4 ± 0.52 (subcutaneous infiltration) vs. 4.9 ± 0.57 (femoral nerve block)). Short time to ambulation (3.4 ± 1.35 h vs. 7.0 ± 2.08 h (subcutaneous infiltration) vs. 4.9 ± 0.32 h (femoral nerve block))	No
Meara et al.^16^ 2011	32 WCI, 33 control Age 5–15 years	Iliac crest bone grafting for alveolar cleft repair	Bupivacaine 0.25% + epinephrine 1: 200.000 Bolus 1.25 mg/kg Continuous 0.75–1 mg/kg/h Duration 24 h	WCI with saline	Wong‐Baker Faces Pain Rating Scale	Maximum difference in pain score at 6 h postoperatively (1.16 ± 0.75 vs. 0.68 ± 0.61), higher pain scores in study group at 18 and 24 h Similar IV morphine use (0.207 ± 0.023 mg/kg vs. 0.224 ± 0.034 mg/kg)	*N* = 1 allergic reaction to dressing
Samartzis et al.^19^ 2016	7 WCI, 5 control Mean age 15.6 years Mean weight 50.5 kg	Posterior spinal fusion with autogenous posterior iliac crest bone grafting	Levobupivacaine Bolus 0.5% 5 mL Continuous 0.25% 3 mL/h Duration 48 h	WCI Bolus 5 mL levobupivacaine 0.5% Continuous infusion of saline	VAS, McGill Pain Questionnaire	Lower pain score in study group (day 1 2.6 ± 2.8 vs. 4.0 ± 3.7, day 2 2.3 ± 2.3 vs. 3.4 ± 2.2) Similar doses of IV morphine (25.7 ± 20.5 mg vs. 20.8 ± 13.0 mg)	*N* = 2 pump obstruction *N* = 1 wound dehiscence
*Extremity surgery*							
Bulut et al.^8^ 2011	20 WCI, 19 control Mean age 6,7 years Mean weight 22,8 kg	Orthopedic and trauma extremity surgery	Bupivacaine 0.5% Bolus 1 mg/kg every 6 h Duration 48 h	WCI with saline	FLACC, Faces Pain Scale	Reduction of pain scores in study group 4‐48 h postoperatively Use of meperidine intramuscularly as rescue medication in 25.0% of study group vs. 73.6% of control group	*N* = 1 spontaneous detachment of WC
Muthusamy et al.^17^ 2010	21 WCI, 16 control Age 4.0–16.2 years Patients with cerebral palsy	Orthopedic extremity surgery: hardware removal postvarus derotational osteotomy, hardware removal postdistal femoral epiphysiodesis, tendo‐achilles lengthening, Strayer”s intramuscular gastrocnemius lengthening	Bupivacaine Bolus 0.2 mg/kg Continuous 0.2 mg/kg/h Duration 48 h	No intervention	Parent Total Quality Pain Management, Noncommunicating Childrenss Pain Checklist‐Postoperative Version	Lower pain score in study group on day 0–2 (day 0 3.79 SD 2.89 vs. 6.70 SD 3.21, day 1 2.78 SD 1.99 vs. 5.17 SD 2.31, day 2 1.79 SD 1.44 vs. 3.43 SD 2.06) Rescue medication mostly oral codeine, in total over 4 days less in study group but not statistically significant (0.68 mg/kg SD 0.66 vs. 1.22 mg/kg SD 1.10)	*N* = 1 kinked catheter causing disruption to the amount of LA infiltrated
*Abdominal surgery*							
Hermanssonet al.^11^ 2013	17 WCI, 15 control Age 0.5–12.6 years Weight 4.7–28.0 kg	Abdominal (enterostomy closure, open gastrostomy, ureteral reimplantation)	Bupivacaine Continuous 0.2–0.4 mg/kg/h Duration 72 h	WCI with saline	FLACC, FPS‐R, VAS	Significantly less opioid consumption in study group (1.71 IV morphine doses vs. 3.81 doses)	*N* = 1 WC was cut accidentally
Machoki et al.^14^ 2015	30 WCI, 17 epidural, 13 control Mean age 6.8 years	Open appendectomy (12 study, 13 control) Laparotomy (18 study, 17 epidural)	Bupivacaine 0.2% Continuous 0.2–0.4 mg/kg/h	Laparotomy patients: epidural Appendectomy patients: no intervention	FLACC	Appendectomy lower pain scores (2.5 SD 0.8 vs. 3.5 SD 0.7) and lower IV morphine requirement (96 SD 50 μg/kg vs. 490 SD 9.4 μg/kg) Laparotomy lower pain scores (2.4 SD 1.2 vs. 3.0 SD 1.2) and lower morphine requirement (230 SD 100 μg/kg, 406 SD 200 μg/kg)	*N* = 4 WCI leak *N* = 1 wound infection in control group

Abbreviations: FLACC, faces legs activity cry consolability behavioral pain scale; FPS‐R, faces pain scale revised; IV, intravenous; LA, local anesthetic, NRS, numerical rating scale; RCT, randomized‐controlled trial; SD, standard deviation; WC, wound catheter; WCI, wound catheter infusion; VAS, visual analogue scale.

#### 3.2.1 Thoracic surgery

Three RCTs investigated WCI as treatment of postoperative pain after thoracic surgery.^9^, ^15^, ^20^

Anell‐Olofsson et al. included 18 preterm infants undergoing ductus ligation via posterolateral thoracotomy.^9^ Mean gestational age (in weeks) at the day of operation was 28 + 3 in the treatment group and 29 + 2 in controls; mean weight at day of operation was 887 g and 840 g, respectively. Infants weighing less than 500 g were excluded, due to limitation of total blood sampling volume. All children received WCI. The study group received a bolus injection of levobupivacaine, followed by a continuous infusion for 8 h. The control group received a bolus and continuous infusion with saline. After the first 8 h and every following 8‐h interval, the two groups switched from levobupivacaine to saline and vice versa. Both groups also received morphine 10 μg/kg/h IV postoperatively and if required, additionally 1 μg/kg fentanyl (IV) as rescue analgesia. The Echelle Douleur Inconfort Nouveau pain score was used, which is validated in this age group.^36^ Median pain scores were nil in both groups (Table 2). The control group, which started with saline infusion, needed more rescue analgesia than the study group, especially in the period 8–16 h postoperatively. The main goal of this study was to test which WCI regimen would provide better analgesia and if plasma concentrations of LA stayed below toxic thresholds.

Mattila et al. investigated 49 patients aged 1–9 years, undergoing atrial septal defect closure via median sternotomy.^15^ Mean age was 4.8 years in the study group and 5.2 years in controls. All children received WCI for 72 h: the study group received ropivacaine, bolus and continuously, the controls received saline. As rescue analgesia, IV morphine was used. The Objective Pain Scale (OPS) was used to measure pain scores, of which we could not find a study assessing its validation.^37^ Primary outcome was the difference in morphine consumption, in which no difference was noticed. Pain scores are presented only in a figure showing the Kaplan–Meier curve for the time point when the OPS was ≥6 for the first time. More than 70% of patients eventually reached a pain score above 6. There were no clinically or statistically significant differences between the groups.

Tirotta et al. studied 72 children >3 months and >5 kg undergoing cardiac surgery via median sternotomy.^20^ Both bupivacaine 0.25% and levobupivacaine 0.25% were used for WCI in the treatment group (Table 2). The control group received WCI with saline. Depending on the patients’ age, different pain scores were used (Faces Legs Activity Cry and Consolability (FLACC), Wong‐Baker FACES Pain Rating Scale, Numeric Rating Scale [NRS]). The FLACC and NRS are validated.^38^, ^39^ Of the Wong‐Baker FACES Pain Rating Scale, validation is reported to be weak.^38^ All children received IV morphine as rescue analgesia; for children >5 years a patient‐controlled analgesia (PCA) system delivering IV morphine was used. The treatment group required clinically and statistically significantly less morphine than the control group. One fifth of patients’ in the treatment group required no morphine at all during the 3 day study period. Pain scores were not different between the groups. One woundcatheter was accidentally removed, this patient's data were withheld from the study.

#### 3.2.2 Iliac crest bone harvesting

Iliac crest bone can be harvested and used as autologous donor bone for several indications, such as cleft alveolar repair or spinal fusion in scoliosis surgery. Harvesting is associated with significant pain at the donor site. Three RCTs investigated WCI at the iliac crest donor site.^13^, ^16^, ^19^

Meara et al. investigated 65 patients 5–15 years of age undergoing iliac crest bone harvesting for cleft alveolar repair.^16^ They used continuous WCI with bupivacaine 0.25% with epinephrine 1:200.000 in the study group. The control group received WCI with saline. The Wong‐Baker Faces Pain Rating Scale was used. The study participants received a PCA with IV morphine as rescue analgesia. There was a small, not clinically or statistically significant difference in total morphine consumption between the groups.

Kumar Raja et al. describe 60 ASA I (American Society of Anesthesiologists Physical Status) patients, with mean age 9.7 years, undergoing iliac crest bone harvesting for cleft alveolar repair.^13^ Patients were divided in three groups, all patients received a wound catheter. Group B received a bolus of bupivacaine via the wound catheter which could be repeated on request postoperatively. This was compared to group A receiving a single shot femoral nerve block and saline boluses WCI on request; and group C receiving one bolus dose of bupivacaine WCI and saline boluses on request. No other rescue medication was used. Pain was scored using the Wong‐Baker Faces Pain Rating Scale. Pain scores were lower in group A and B, compared to group C. Time to ambulation was longer in group A and C, compared to group B. In this study, there are concerns regarding the blinding of the patients and outcome assessors, because patients in group A had a motor nerve blockade of the leg.

In Samartzis et al study, 12 patients weighing >35 kg and aged 10–18 years were observed undergoing posterior iliac crest bone harvesting for posterior spinal fusion.^19^ WCI with levobupivacaine was used in bolus and continuously (Table 2). The control group received WCI with a similar bolus of levobupivacaine, followed by a continuous infusion of saline. The Visual Analogue Scale (VAS) and McGill Pain Questionnaires were used to assess pain. The VAS is weakly validated in children, the McGill Pain Questionnaire is only validated in adults.^38^, ^40^ Pain scores were examined at the donor site and the contralateral iliac crest bone site. It was not described why pain on the contralateral site was tested. As rescue analgesia, PCA morphine IV was provided. VAS pain scores were lower in the study group than in the control group, no clinically or statistically significant difference was shown. There was no difference in use of rescue morphine. Wound dehiscence occurred in one patient in the control group, two patients’ data were withheld from the study due to obstruction of the wound catheter.

#### 3.2.3 Extremity surgery

Two RCTs investigated WCI after extremity surgery.^8^, ^17^

In Bulut et al., 39 patients undergoing various elective orthopedic or traumatologic extremity procedures were studied.^8^ Patients aged between 1 and 12 years and ASA I–II were included. WCI with bupivacaine was used, in boluses, which were repeated every 6 h (Table 2). The control group received WCI with boluses of saline every 6 h. Meperidine (0.75 mg/kg intramuscularly) was used as rescue medication. The FLACC and Faces Pain Scale were used.^39^ Pain scores were clinically and statistically significantly lower in the study group. The pain scores were only shown in a figure, numerical outcomes were not provided. In the study group, 5 patients received meperidine, compared to 14 patients in the control group.

Muthusamy et al. studied 37 children with cerebral palsy, aged 3 to 18 years, undergoing various orthopedic surgical procedures on the lower extremity. The surgeries performed were comparable between the two groups.^17^ The study group received WCI with a bolus and continuous infusion of bupivacaine. The patients in the control group did not receive a wound catheter, therefore this study was not blinded. Both groups received oral analgesics, preferably paracetamol and codeine, as rescue medication. The parents removed the catheter after 48 h. Parents reported a VAS score representing the daily level of pain severity. Furthermore, they filled in the Parent Total Quality Pain Management and Noncommunicating Children's Pain Checklist‐Postoperative Version; which are both validated and the latter is designed for evaluating pain in children with intellectual disabilities.^41^ Clinically significantly lower pain scores were seen in the study group, with a statistically significant difference on day 0 and day 1. Parents reported they were satisfied (23.3%) to very satisfied (73.3%) with the postoperative pain treatment. One patient developed a kink in the wound catheter, causing disruption to the amount of LA infiltrated in the wound.

#### 3.2.4 Abdominal surgery

Two RCTs were identified regarding WCI after abdominal surgery.^11^, ^14^

Hermansson et al. studied 32 children (6 months–13 years) undergoing elective abdominal surgery, including open gastrostomy, enterostomy closure or urethral reimplantation.^11^ Intraoperatively, fentanyl was administered. WCI with bupivacaine was used in the treatment group, the control group received WCI with saline. As rescue analgesia, IV morphine was used. Primary outcome was total dose of morphine, which showed to be clinically and statistically significantly lower on the first postoperative day in the bupivacaine group. However, on day 2 and 3 postoperatively, no difference was seen. Pain scores were not reported.

Machoki et al. evaluated 60 patients, aged between 3 months and 12 years old.^14^ Of these, 25 underwent open appendectomy and received either WCI with bupivacaine or only systemic pain medication. The other 35 underwent a laparotomy and were randomized into receiving analgesia via WCI with bupivacaine or via an epidural catheter. The patients were not blinded for the treatment. The FLACC pain score was used for all patients, although this score is only validated for patients in a young age range.^39^ In all groups, IV morphine was used as rescue analgesia. Overall, a difference favoring the use of WCI is suggested, with lower pain scores and opioid consumption in the study groups. However, the pain scores did not show a statistical difference. Morphine requirement was 10‐fold higher in patients undergoing appendectomy without WCI versus those with WCI, which was a statistically significant difference.

### 3.3 Adverse events

To analyze adverse events, we included all 28 relevant original studies regarding WCI in children. These also included observational studies, retrospective studies, case‐series and case‐reports on infants, children and adolescents undergoing thoracic surgery, iliac crest bone harvesting, extremity surgery, abdominal surgery, major urology surgery and costal cartilage harvesting. Included were 712 study patients undergoing 714 operations, with WCI with LA as treatment of postoperative pain; 762 patients were included as controls. Of these control patients, 200 received WCI with saline or another infusion regimen. The other 562 patients received different types of analgesia (epidural, intercostal, systemic opioids, or only rescue medication).

In all WCI patients receiving LA, no clinical symptoms associated with LAST were reported. Two patients in the group receiving LA developed a wound infection^9^, ^26^ and one developed an infected seroma at the site of the wound.^34^ In one patient, neuropathic pain occurred at the wound site.^32^ It was described as a mild intensity burning with dysesthesia and tingling and did not require pharmacological treatment. Wound dehiscence occurred once (control group, saline via WCI).^19^ An unexpected loosening of one of the skin sutures was described once.^26^ One patient had an allergic reaction to the adhesive bandage applied over the woundcatheter.^16^ There were 19 patients (treatment or control group) who experienced any catheter‐related adverse events like obstruction or leakage.

### 3.4 Pharmacokinetic testing

Of all 28 studies, five studies measured plasma concentrations of LA (Table 3).^9^, ^10^, ^18^, ^20^, ^26^ In the study by Anell‐Olofsson et al., plasma concentrations of levobupivacaine remained below the threshold for systemic toxicity of 2 μg/mL.^9^, ^42^ In one sample, a concentration of 1.6 μg/mL was measured, advancing the threshold. Krylborn et al. observed 20 children with a mean age of 4 days old undergoing major thoracic or abdominal surgery and measured plasma levels of levobupivacaine (both total and unbound levobupivacaine), throughout 72 h of continuous infusion.^26^ The authors state that the plasma toxicity threshold for levobupivacaine is unknown, therefore the plasma concentrations were compared to the toxic threshold of bupivacaine; these thresholds were not exceeded. In the study by Tirotta et al., plasma concentrations of levobupivacaine were measured.^20^ The authors state that all plasma concentrations stayed below the toxic threshold, however they mention a threshold of 4 μg/mL.

**TABLE 3:** Plasma concentrations of local anesthetics during wound catheter infusion.

Study reference	Local anesthetic used	Maximum plasma concentration	Symptoms of local anesthetic systemic toxicity
Anell‐Olofsson et al.^9^	Levobupivacaine	1.682 μg/mL^a^	No
Hayes et al.^10^	Bupivacaine	0.7 ± 0.6 μg/mL^b^	No
Krylborn et al.^26^	Levobupivacaine	3.15 μg/mL^a^ 0.21 μg/mL^c^	No
Niiyama et al.^18^	Ropivacaine	0.9 ± 0.5 μg/mL^b^	No
Tirotta et al.^20^	Levobupivacaine	± 2.5 μg/mL^b^, ^d^	No

*Note*: For more details regarding admission regimens of local anesthetics, see Table 2. Some studies report the absolute number of the highest plasma concentration measured,^9^, ^26^ others report a mean ± standard deviation of the highest plasma concentrations measured.^10^, ^18^

*Note*: Plasma toxicity thresholds. Bupivacaine: 4 μg/mL (total),^43^ 0.12 μg/mL (unbound^42^). Levobupivacaine: 2 μg/mL (total),^42^ unbound concentration unknown. Ropivacaine 2.3 μg/mL (total),^43^ 0.16 μg/mL (unbound).^42^

^a^ Total plasma concentration.

^b^ Article does not mention if plasma concentration is of total, bound or unbound fraction.

^c^ Unbound plasma concentration.

^d^ This is an estimation from the highest point in a graph, as this was the only way the measurements of plasma concentrations were presented in the article.

Hayes et al. investigated 54 children and adolescents (mean age 12 years) undergoing iliac crest bone harvesting, using WCI with bupivacaine.^10^ Plasma levels remained below the toxic threshold of 4 μg/mL.^43^ One child accidentally received a bolus of 50 mL of bupivacaine 0.25% due to a pump programming error. No clinical symptoms of toxicity were noticed. The specific plasma concentration of this individual is not provided.

Niiyama et al. investigated 48 children with a mean age of 11.4 years, receiving ropivacaine for costal cartilage harvesting.^18^ None of the blood samples surpassed the toxic threshold of 2.3 μg/mL.^18^, ^42^

## 4 DISCUSSION

This systematic review was conducted to assess the efficacy and safety of WCI with LA for the treatment of postoperative pain in children. Ten RCTs of good or excellent quality were found. Only studies on postoperative WCI with LA after extremity and abdominal surgery demonstrated effective postoperative analgesia and/or an opioid sparing effect, therefore this technique may be considered as treatment of postoperative pain in these indications.^8^, ^11^, ^14^, ^17^

In 28 studies, no serious adverse events, such as LAST, regarding the use of WCI with LA occurred, and five investigations on plasma concentrations of LA did not show toxic concentrations. Some adverse effects, such as infection, were mentioned, however the incidence was low. Therefore, the use of WCI with LA may be regarded as a safe technique in children.

The major limitation of this review is that 10 RCTs of good or excellent quality were found, investigating children in different age groups, undergoing different types of operations. Different types of postoperative pain treatment were used as comparison, and different methods of rescue analgesia. Due to this heterogeneity, results could not be pooled, and therefore it is difficult to compare the efficacy between techniques.

Even in the RCTs of good or excellent quality, there were occasionally concerns regarding the methodology, such as blinding to the treatment. Furthermore, when investigating pain in pediatric patients, it is important to use age‐appropriate pain assessment instruments. Only four out of 10 RCTs in this review used pain scales, validated for the specific age group investigated.^8^, ^9^, ^17^, ^20^ One study investigated children with cerebral palsy.^17^ Although validated questionnaires were used, it is still more difficult to assess pain in patients with impairment.

Although this may be a general concern in pediatric pain research, this finding emphasizes the need for more high quality investigations on the efficacy of WCI (or any RA technique) in pediatric anesthesia, also comparing this technique with current standard of care, such as neuraxial analgesia or peripheral nerve blocks.

Reducing the need for opioids, may be beneficial due to its effect on respiratory function, especially in young infants, and on side effects like vomiting, urinary retention and sedation levels.^44^, ^45^ Not in all studies an opioid sparing effect of WCI could be demonstrated. However, some studies included a standard continuous infusion of opioids for study and control patients. This could have interfered with the results. Preferably, opioids should be administered on request, if possible, to more adequately measure the effect of RA.

Systematic reviews in adults find that WCI is non‐inferior to epidural analgesia.^1^, ^46^ Although performed under general anesthesia or sedation, neuraxial analgesia is considered safe, even in young children.^3^, ^4^, ^5^ Still, in neuraxial blocks complications such as dural puncture, transient neurological deficits or epidural abcess may occur.^4^, ^47^ WCI could provide effective treatment of pain without those risks associated with neuraxial techniques. Also, epidural catheter placement is not always successful: in a retrospective study in 90 children (mean age 3.7 years) receiving epidural analgesia after abdominal surgery, in 35% epidural analgesia was considered not succesfull, and catheters had to be removed because of technical failure or inadequate analgesia.^48^ As the woundcatheter is placed under direct vision, this may be an advantage. Another possible advantage of WCI is that patients could be discharged home with WCI using elastomeric bulb devices.^17^ If WCI is used in an outpatient setting for selected types of procedures, it could contribute to a more efficient allocation of scarce health care means.

In this review, out of 912 patients receiving either LA or saline via WCI, two developed a wound infection and one an infected seroma, not leading to any long‐lasting effects.^9^, ^26^, ^34^ In vitro, LAs inhibit the growth of a spectrum of bacterial pathogens common in surgical site infections, thereby possibly having a protective effect on the occurrence of wound infections.^49^Adverse events indicating LAST during WCI were not found. Most studies have been performed with bupivacaine as LA. However, levobupivacaine and ropivacaine are associated with a lower risk for LAST compared to bupivacaine, and should therefore preferably be used in children.^50^ A concern could be that young children are not able to complain about mild LAST symptoms, such as tinnitus or altered taste sensation. Therefore, regarding young children, we can only state that more serious LAST effects were not noticed (convulsions, arrhythmia).

Amide LA (such as (levo)bupivacaine and ropivacaine) bind to serum AGP (α1‐acid glycoprotein).^51^ AGP concentrations are very low at birth and progressively increase during the first year of life. This is why neonates and young infants have a much higher free fraction of LA than adults.^51^ Also, CYP1A2 activity, which metabolizes ropivacaine and, partly, levobupivacaine, is immature before the age of 4–7 years.^51^ Young children are therefore more prone to develop LAST. Although WCI administers LA continuously in richly vascular tissues, plasma concentrations of LA during continuous WCI were only investigated in five studies in pediatric patients, of which only two investigated infants.^9^, ^10^, ^18^, ^20^, ^26^

In conclusion, in the few studies we found, the use of WCI with LA for postoperative analgesia in children showed to be an effective treatment of postoperative pain after abdominal and extremity surgery. No serious adverse events, such as of LAST, were reported from preterm children to adolescents.

## AUTHOR CONTRIBUTIONS

**Dominique J. Swenker**: This author was involved in the design of the study, retrieving the reports of the updated search in 2020, analysis and interpretation of the data, drafting the manuscript and revising it critically for important intellectual content. She approved the final version to be published and agreed to be accountable for all aspects of the work. **Maaike Dirckx**: This author was involved in the design of the study, analysis and interpretation of the data, drafting the manuscript and revising it critically for important intellectual content. She approved the final version to be published and agreed to be accountable for all aspects of the work. **Lonneke M. Staals**: This author was involved in the design of the study, retrieving the reports of the search in 2015, analysis and interpretation of the data, drafting the manuscript and revising it critically for important intellectual content. She approved the final version to be published and agreed to be accountable for all aspects of the work.

## FUNDING INFORMATION

This work was funded by departmental funding.

## CONFLICT OF INTEREST STATEMENT

The authors have no conflicts of interest.

## APPENDIX 1

### SEARCH STRATEGY

Date search: 5th of February 2015.

Date update of search: 31st of July 2020.

Sources: Embase, Medline (Ovid‐SP), Cochrane Central, Web of Science, PubMed recent.

Embase, children: 809, updated search 1133.

(“postoperative pain”/de OR “Postoperative analgesia”/de OR ((Postoper* OR operat* OR surgic* OR surger*) NEAR/6 (pain* OR analges*)):ab,ti) AND (“Local Anesthesia”/de OR “Local anesthetic agent”/exp OR (((local* OR infiltr*) NEAR/3 (anesth* OR anaesth*)) OR bupivacain* OR levobupivacain* OR lidocain* OR ropivacain*):ab,ti) AND (“Continuous infusion”/de OR ((Catheter/de OR (catheter*):ab,ti) AND (“surgical wound”/de OR ((operat* OR surg*) NEAR/3 wound*):ab,ti)) OR (infus* OR infiltrat* OR (wound* NEAR/3 cathet*) OR SPWC):ab,ti) AND (child/exp OR newborn/exp OR adolescent/exp OR adolescence/exp OR pediatrics/exp OR childhood/exp OR “child health”/de OR “child health care”/exp OR “childhood disease”/exp OR “child death”/de OR “pediatric ward”/de OR “pediatric hospital”/de OR “pediatric surgery”/exp OR (adolescen* OR infan* OR newborn* OR (new NEXT/1 born*) OR baby OR babies OR neonat* OR child OR children OR childhood OR kid OR kids OR toddler* OR teen* OR boy* OR girl* OR minors OR underag* OR (under NEXT/1 (age* OR aging)) OR juvenil* OR youth* OR puber* OR pubescen* OR prepubescen* OR prepubert* OR pediatric* OR pediatric*):ab,ti) NOT ([animals]/lim NOT [humans]/lim).

Medline (Ovid‐SP): 684, updated search 899.

(“Pain, Postoperative”/ OR ((Postoper* OR operat* OR surgic* OR surger*) ADJ6 (pain* OR analges*)).ab, ti.) AND (“Anesthesia, Local”/ OR exp “Anesthetics, Local”/ OR (((local* OR infiltr*) ADJ3 (anesth* OR anaesth*)) OR bupivacaine* OR levobupivacain* OR lidocain* OR ropivacain*).ab, ti.) AND (“Catheters”/ OR (infus* OR infiltrat* OR (wound* ADJ3 cathet*) OR SPWC).ab, ti.) AND (exp “child”/ OR exp “infant”/ OR exp “adolescent”/ OR exp “pediatrics”/ OR exp “child care”/ OR exp “hospitals, pediatric”/ OR (adolescen* OR infan* OR newborn* OR (new ADJ born*) OR baby OR babies OR neonat* OR child OR children OR childhood OR kid OR kids OR toddler* OR teen* OR boy* OR girl* OR minors OR underag* OR (under ADJ (age* OR aging)) OR juvenil* OR youth* OR puber* OR pubescen* OR prepubescen* OR prepubert* OR pediatric* OR pediatric*).ab, ti.) NOT (animals NOT humans).sh.

Cochrane Central (trials): 175, updated search 419.

((Postoper* OR operat* OR surgic* OR surger*) NEAR/6 (pain* OR analges*)):ab, ti AND (((local* OR infiltr*) NEAR/3 (anesth* OR anaesth*)) OR bupivacain* OR levobupivacain* OR lidocain* OR ropivacain*):ab, ti AND ((catheter* AND ((operat* OR surg*) NEAR/3 wound*)) OR infus* OR infiltrat* OR (wound* NEAR/3 cathet*) OR SPWC):ab, ti AND ((adolescen* OR infan* OR newborn* OR (new NEXT/1 born*) OR baby OR babies OR neonat* OR child OR children OR childhood OR kid OR kids OR toddler* OR teen* OR boy* OR girl* OR minors OR underag* OR (under NEXT/1 (age* OR aging)) OR juvenil* OR youth* OR puber* OR pubescen* OR prepubescen* OR prepubert* OR pediatric* OR pediatric*):ab, ti).

Web of Science: 545, updated search 744.

TS = (((Postoper* OR operat* OR surgic* OR surger*) NEAR/6 (pain* OR analges*)) AND (((local* OR infiltr*) NEAR/3 (anesth* OR anaesth*)) OR bupivacain* OR levobupivacain* OR lidocain* OR ropivacain*) AND ((catheter* AND ((operat* OR surg*) NEAR/3 wound*)) OR infus* OR infiltrat* OR (wound* NEAR/3 cathet*) OR SPWC) AND ((adolescen* OR infan* OR newborn* OR (new NEXT/1 born*) OR baby OR babies OR neonat* OR child OR children OR childhood OR kid OR kids OR toddler* OR teen* OR boy* OR girl* OR minors OR underag* OR (under NEXT/1 (age* OR aging)) OR juvenil* OR youth* OR puber* OR pubescen* OR prepubescen* OR prepubert* OR pediatric* OR pediatric*)) NOT ((animal* OR pig*) NOT (human* OR patient*))).

PubMed recent (as supplied by publisher): 3.

The updated search was not conducted in PubMed recent, because in 2020 these references will also be found using Medline (Ovid‐SP).

((Postoper*[tiab] OR operat*[tiab] OR surgic*[tiab] OR surger*[tiab]) AND (pain*[tiab] OR analges*[tiab])) AND (((local*[tiab] OR infiltr*[tiab]) NEAR/3 (anesth*[tiab] OR anaesth*[tiab])) OR bupivacain*[tiab] OR levobupivacain*[tiab] OR lidocain*[tiab] OR ropivacain*[tiab]) AND ((catheter*[tiab] AND ((operat*[tiab] OR surg*[tiab]) NEAR/3 wound*[tiab])) OR infus*[tiab] OR infiltrat*[tiab] OR (wound*[tiab] NEAR/3 cathet*[tiab]) OR SPWC[tiab]) AND ((adolescen*[tiab] OR infan*[tiab] OR newborn*[tiab] OR (new born*[tiab]) OR baby[tiab] OR babies[tiab] OR neonat*[tiab] OR child[tiab] OR children[tiab] OR childhood[tiab] OR kid[tiab] OR kids[tiab] OR toddler*[tiab] OR teen*[tiab] OR boy*[tiab] OR girl*[tiab] OR minors[tiab] OR underag*[tiab] OR under age*[tiab] OR under aging[tiab] OR juvenil*[tiab] OR youth*[tiab] OR puber*[tiab] OR pubescen*[tiab] OR prepubescen*[tiab] OR prepubert*[tiab] OR pediatric*[tiab] OR pediatric*[tiab])) AND publisher[sb].
